# Supplementary material for: Experimental evidence of subtle victim blame in the absence of explicit blame
Source: PLoS One. 2019 Dec 30;14(12):e0227229. doi: 10.1371/journal.pone.0227229 (PMC6936882; doi:10.1371/journal.pone.0227229)
Supplement: S1 Table — (DOCX) [file pone.0227229.s001.docx]

**S1 Table. Hierarchical regression with self-deceptive enhancement as the measure of socially desirable responding, Study 2.**

| Predictor | *b* | *t* | *p* | *sr*2 | 95% CI of *b* |
| --- | --- | --- | --- | --- | --- |
| Step 1: *R*^2^ = .19, *F*(4, 290) = 17.12, *p* < .001 | | |  |  |  |
| Self-deceptive enhancement (SDE) | -0.46 | -1.77 | .08 | .01 | [-0.97, 0.05] |
| Victim suffering (VS) | 0.08 | 0.74 | .46 | .002 | [-0.13, 0.29] |
| Low control behaviors | 0.09 | 2.35 | .02 | .02 | [0.02, 0.17] |
| High control behaviors/subtle blame (SB) | 0.31 | 7.11 | < .001 | .14 | [0.22, 0.40] |
| Step 2: *R*^2^_change_ = .01, *F*_change_(3, 287) = 1.56, *p* = .20 | | |  |  |  |
| SDE X VS | -0.56 | -1.07 | .28 | .003 | [-1.59, 0.47] |
| SDE X SB | -0.34 | -1.77 | .08 | .01 | [-0.73, 0.04] |
| VS X SB | 0.02 | 0.18 | .86 | < .001 | [-0.15, 0.19] |
| Step 3: *R*^2^_change_ = .01, *F*_change_(1, 286) = 4.01, *p* = .046 | | |  |  |  |
| SDE X VS X SB | -0.77 | -2.00 | .046 | .01 | [-1.53, -0.01] |

Criterion = explicit blame. Victim suffering = severe suffering (1) vs. mild suffering (0).
