# Supplementary material for: Experimental evidence of subtle victim blame in the absence of explicit blame
Source: PLoS One. 2019 Dec 30;14(12):e0227229. doi: 10.1371/journal.pone.0227229 (PMC6936882; doi:10.1371/journal.pone.0227229)
Supplement: S2 Table — (DOCX) [file pone.0227229.s002.docx]

**S2 Table. Hierarchical regression with impression management as the measure of socially desirable responding, Study 2.**

| Predictor | *b* | *t* | *p* | *sr*2 | 95% CI of *b* |
| --- | --- | --- | --- | --- | --- |
| Step 1: *R*^2^ = .19, *F*(4, 290) = 17.00, *p* < .001 | | |  |  |  |
| Impression management (IM) | -0.39 | -1.66 | .099 | .01 | [-0.85, 0.07] |
| Victim suffering (VS) | 0.08 | 0.79 | .43 | .002 | [-0.13, 0.29] |
| Low control behaviors | 0.10 | 2.44 | .02 | .02 | [0.02, 0.17] |
| High control behaviors/subtle blame (SB) | 0.31 | 7.05 | < .001 | .14 | [0.22, 0.39] |
| Step 2: *R*^2^_change_ = .01, *F*_change_(3, 287) = 0.57, *p* = .64 | | |  |  |  |
| IM X VS | -0.32 | -0.66 | .51 | .001 | [-1.27, 0.63] |
| IM X SB | -0.17 | -0.92 | .36 | .002 | [-0.53, 0.19] |
| VS X SB | 0.04 | 0.44 | .66 | < .001 | [-0.13, 0.21] |
| Step 3: *R*^2^_change_ = .004, *F*_change_(1, 286) = 1.44, *p* = .23 | | |  |  |  |
| IM X VS X SB | -0.46 | -1.20 | .23 | .004 | [-1.22, 0.30] |

Criterion = explicit blame. Victim suffering = severe suffering (1) vs. mild suffering (0).
