# Supplementary material for: Experimental evidence of subtle victim blame in the absence of explicit blame
Source: PLoS One. 2019 Dec 30;14(12):e0227229. doi: 10.1371/journal.pone.0227229 (PMC6936882; doi:10.1371/journal.pone.0227229)
Supplement: S2 Text — (DOCX) [file pone.0227229.s004.docx]

**S2 Text. Mixed ANOVA, Study 2**

We conducted a Victim Suffering (mild vs. severe) X Controllability of Causes (low control vs. high control behaviors) mixed ANOVA with repeated measures on the second factor. The dependent variable was the perceived likelihood that the behaviors caused the victim’s sepsis. The low control behaviors were seen as more likely to have caused the sepsis than the high control behaviors (*M*s = 3.71 vs. 2.97, respectively), *F*(1, 293) = 56.94, *p* < .001, η*_p_*^2^ = .16. There was no main effect for victim suffering, *F*(1, 293) = 0.22, *p* = .64, η*_p_*^2^ = .001.

There was, however, a Victim Suffering X Controllability of Causes interaction, *F*(1, 293) = 6.73, *p* = .01, η*_p_*^2^ = .02. LSD comparisons showed that, consistent with our reasoning, participants in the severe suffering condition rated the high control behaviors as more likely to have caused the victim’s sepsis than did participants in the mild suffering condition, *p* = .03, *d* = 0.24, 95% CI [0.03, 0.59]. Ratings for the low control behaviors did not differ by condition, *p* = .21, *d* = -0.15, 95% CI [-0.51, 0.11].
