# Supplementary material for: Experimental evidence of subtle victim blame in the absence of explicit blame
Source: PLoS One. 2019 Dec 30;14(12):e0227229. doi: 10.1371/journal.pone.0227229 (PMC6936882; doi:10.1371/journal.pone.0227229)
Supplement: Attachment — Submitted filename: Response to Reviewers.docx [file pone.0227229.s005.docx]

Dear Dr. Wisneski,

Thank-you for the opportunity to revise and resubmit our manuscript. The reviewers’ comments were very helpful. Please see below for explanations regarding how we dealt with each of the reviewer’s points.

We look forward to hearing from you soon.

Sincerely,

Carolyn Hafer

**Reviewer #1**

1) - Effect of suffering on attributions to low and high control causes in Studies 1 and 2. I appreciate the appropriateness of ANCOVA given hypothesis 1. It would be informative to show also whether the effect of suffering on low and high control causes differs in the way that the ANCOVA’s (on high control) and t-test’s (low control) might imply, but do not confirm – i.e., whether there is an interaction such that suffering increases attributions to high (compared to low) control causes more strongly. The results clearly suggest that greater suffering motivated ppts. to find fault with the victim’s behavior in particular, rather than more strongly endorse any old cause. If suffering does increase high control attributions significantly over and above low control attributions, I think a stronger conclusion can be drawn. These additional analyses could be reported briefly in a footnote or supplementary materials.

**Our response:** The analysis suggested by Reviewer #1 is a Victim Suffering (mild vs. severe) X Controllability of Causes (low control vs. high control behaviors) mixed ANOVA with repeated measures on the second factor. The dependent variable is the perceived likelihood that the behaviors caused the victim’s sepsis. We conducted this mixed ANOVA for Studies 1 and 2. In both cases, there is a significant interaction between victim suffering and controllability of causes: Participants in the severe suffering condition rated the high control behaviors as more likely to have caused the victim’s sepsis than did participants in the mild suffering condition, whereas ratings for the low control behaviors did not differ by condition. Thus, as Reviewer #1 suggests should be the case, greater suffering increases high control attributions significantly, independent of any such effect on low control attributions. In the revised manuscript, we briefly state the findings of these mixed ANOVAs in the first paragraph of the “Victim blame” section of the Results for Studies 1 and 2. We refer to new supplemental information files (S3_Mixed ANOVA, S4_Mixed ANOVA) for details.

2) - Self-deception vs. impression management. In Study 2, the relation between implicit and explicit blame was moderated (under high suffering) by the self-deception, but not the impression management subscale, of the BIDR. The pattern of results supports the idea that people resort to subtle over overt blame due to social desirability concerns in general. Yet, might this pattern also suggest something about the specific locus of these concerns? My understanding is that the self-deception subscale measures the tendency to maintain a positive self-image (I don’t want to see myself as counter-normative/a person who blames victims), whereas the impression management subscale pertains to managing others’ impressions of the self (I don’t want to be seen by others as behaving counter-normatively). Space permitting, I think it would be good to briefly address this.

**Our response:**  Reviewer 1’s point is related to our comment at the end of the “Limitations and future directions” section regarding the conscious versus unconscious nature of subtle blame. Thus, we added a sentence to this paragraph stating one implication of our finding that *only* self-deceptive enhancement moderated the relation between subtle and explicit blame.

3) - It would be helpful to provide intercorrelations between measures in Study 1

**Our response:** We revised Table 1 so that it now contains intercorrelations for Study 1 and Study 2. We also moved Table 1 to immediately after its first mention (the end of the 1^st^ paragraph for the Results and Discussion of Study 1).

4) - There is a (presumed) typo on line 177 (data collection, rather than analysis?)

**Our response:** The Reviewer is correct. We have changed “data analysis” to “data collection” (see the 1^st^ paragraph of Study 1).

5) - A reference is needed on line 384 (research on relation between explicit/implicit constructs)

**Our response:** We have added a citation (see 2^nd^ paragraph of the Discussion for Study 2).

**Reviewer #2**

1) I think some concrete examples of high vs. low control causes might help provide the reader with a bit more context for the work. Somewhere around line 83.

**Our response:** We have included two examples of high and low control causes (see the end of the 4^th^ paragraph of the Introduction).

2) Lerner’s theorizing about the two forms of the justice motive—basically, implicit vs. explicit--doesn’t seem to be given much coverage here. It might not be exactly relevant to the current work, but mention of this theorizing might provide a bit more theoretical grounding for the research.

**Our response:** We have added Lerner’s notion of deliberative/rational versus intuitive/defensive processes with respect to the justice motive (see the 3^rd^ paragraph of the Introduction).

3) I wondered whether it would have been more appropriate to control for high control variables when testing the effect of suffering status on low control behaviors (as was done for low control behaviors)

**Our response:** We have replaced the *t* tests using low control behaviors as the dependent variable with ANCOVAs, as Reviewer #2 suggests (see the 1^st^ paragraph of the “Victim blame” section of the Results for Studies 1 and 2).

4) Most of the theorizing in the introduction about the role of SDR was in general terms but the authors analysed impression management and self-deception separately. I think this is fine given the known two subcomponents of the BIDR, but it would be good to see more discussion of why the effects were observed for SDE and not impression management. What are the differences between these forms of SDR that might shed light on why effects were observed for one and not the other?

**Our response:** See our response to Reviewer #1’s Point 2.
